# Surgical Trial in Lobar Intracerebral Haemorrhage (STICH II) Protocol

**DOI:** 10.1186/1745-6215-12-124

**Published:** 2011-05-17

**Authors:** A David Mendelow, Barbara A Gregson, Patrick M Mitchell, Gordon D Murray, Elise N Rowan, Anil R Gholkar

**Affiliations:** 1 STICH Office, Neurosurgical Trials Unit, Newcastle University, 3-4 Claremont Terrace, Newcastle upon Tyne, NE2 4AE, UK; 2 Centre for Population Health Sciences, University of Edinburgh Medical School, Teviot Place, Edinburgh EH8 9AG, UK; 3 Regional Neurosciences Centre, The Newcastle upon Tyne Hospitals NHS Foundation Trust, Royal Victoria Infirmary, Queen Victoria Road, Newcastle upon Tyne, NE1 4LP, UK

## Abstract

**Background:**

Within the spectrum of spontaneous intracerebral haemorrhage there are some patients with large or space occupying haemorrhage who require surgery for neurological deterioration and others with small haematomas who should be managed conservatively. There is equipoise about the management of patients between these two extremes. In particular there is some evidence that patients with lobar haematomas and no intraventricular haemorrhage might benefit from haematoma evacuation. The STICH II study will establish whether a policy of earlier surgical evacuation of the haematoma in selected patients will improve outcome compared to a policy of initial conservative treatment.

**Methods/Design:**

an international multicentre randomised parallel group trial. Only patients for whom the treating neurosurgeon is in equipoise about the benefits of early craniotomy compared to initial conservative treatment are eligible. All patients must have a CT scan confirming spontaneous lobar intracerebral haemorrhage (≤1 cm from the cortex surface of the brain and 10-100 ml in volume). Any clotting or coagulation problems must be corrected and randomisation must take place within 48 hours of ictus. With 600 patients, the study will be able to demonstrate a 12% benefit from surgery (2p < 0.05) with 80% power.

Stratified randomisation is undertaken using a central 24 hour randomisation service accessed by telephone or web. Patients randomised to early surgery should have the operation within 12 hours. Information about the status (Glasgow Coma Score and focal signs) of all patients through the first five days of their trial progress is also collected in addition to another CT scan at about five days (+/- 2 days). Outcome is measured at six months via a postal questionnaire to the patient. Primary outcome is death or severe disability defined using a prognosis based 8 point Glasgow Outcome Scale. Secondary outcomes include: Mortality, Rankin, Barthel, EuroQol, and Survival.

**Trial Registration:**

ISRCTN: ISRCTN22153967

## Trial Hypothesis

A policy of earlier surgical evacuation of the haematoma in selected patients with spontaneous lobar ICH improves outcome compared to a policy of initial conservative treatment.

## Background

### The role of operative neurosurgical intervention in intracerebral haemorrhage is controversial

Spontaneous intracerebral haemorrhage (ICH) accounts for 10 to 40% of all cases of stroke (there is some variation between countries) and is common in younger patients [1]. The morbidity and mortality exceed 60% and young disabled survivors are a significant burden to both Health and Social Services with only 12% of all ICH patients emerging with minor handicap [2]. The role of operative neurosurgical intervention is controversial and the practice continues to be haphazard [3,4]. Within the spectrum of ICH there are some patients (with large or space occupying ICH) who require surgery for neurological deterioration and others with small haematomas who should be managed conservatively. There is equipoise about the management of patients between these two extremes. Some patients have a penumbra of functionally impaired but potentially viable tissue around the ICH. Surgical removal of the clot may improve the function and recovery in this penumbra [5].

### Findings from previous randomised trials have not been significant

The first randomised trial of Surgical Treatment of ICH, published in 1961 [6] did not show a significant advantage for either surgical or conservative treatment. However this trial was prior to CT and modern operative techniques and care facilities. Between 1989 and 1992 results from four small prospective randomised trials were published. Two trials showed a non-significant advantage for surgery [7,8] and two favoured conservative treatment but the advantage was not significant [9,10]. Two further very small trials have been published both showing a non-significant advantage in favour of surgery [11,12]. Each of these reported problems with recruiting sufficient patients from a single centre and argued for the importance of a large randomised multicentre trial. Further trials have reported since 2000: a large trial of 500 patients showing a non-significant advantage for surgery [13]; two smaller trials showing a significant advantage for surgery [14,15] and a small trial suggesting an advantage for conservative treatment [16]. The need to gain robust evidence to support clinical decision making led to the initiation of the Surgical Trial in Intracerebral Haemorrhage (STICH). The funding for STICH was provided from the UK by the MRC and the Stroke Association and was activated in 1998 and 1995 respectively. This trial is the largest to date and successfully recruited 1033 patients from 87 centres around the world. It also suggested a small non-significant advantage for surgery [17].

### Meta-analyses suggest that surgery may benefit a sub group of patients

**Figure 1:**
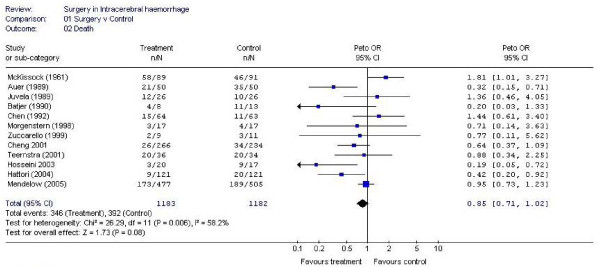
**Meta-analysis of all surgical intracerebral haemorrhage trials (Poor outcome = death)**.

A meta-analysis of the first four published randomised controlled trials was conducted by Prasad et al (2000) for the Cochrane Collaboration [18]. This was updated in 2006 (see figure 1) to include all twelve trials published prior to 2006. Including all twelve trials gave an odds ratio of 0.85(CI 0.71, 1.02) in favour of surgical treatment when the unfavourable outcome was death and an odds ratio of 0.86 (CI 0.72, 1.03) for the 11 trials with published data when the unfavourable outcome was severe disability or death.

Further detailed analysis of the CT images showed that 42% of patients included in STICH who had assessable scans also had an associated intraventricular haemorrhage (IVH). The prognosis for patients with intraventricular haemorrhage with or without hydrocephalus is much worse than that for intracerebral haemorrhage alone. Removing these patients from the analysis and focusing on superficial haematomas presented a more encouraging picture for surgery. There were 223 patients in STICH with such haematomas and with initial conservative treatment 37% achieved a favourable outcome using the prognosis based outcome methodology used in STICH [19]. By contrast 49% of patients achieved a favourable outcome with early surgery (p = 0.080). Furthermore using prognosis based Rankin as the outcome variable a significant benefit was observed for surgical patients in this subgroup. (p = 0.013). Although this is a post hoc identified subgroup, the exclusion of IVH makes clinical sense in the context of debulking surgery for lobar haematomas. The treatment of IVH is different and does not involve craniotomy.

**Figure 2:**
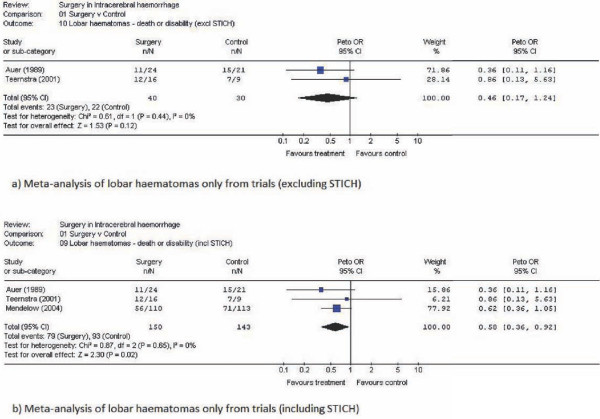
**Meta-analysis of lobar haematomas only; a) excluding STICH, b) including STICH**.

The majority of patients in the other trials reported in the meta-analysis had deep haematomas. Only in the trials by Auer et al. (45 patients) and Teernstra et al. (23 patients) [7,16] did the numbers with lobar ICH reach double figures. In the Auer et al. trial 54% of the 24 surgical patients had a favourable outcome compared to 29% of the 21 conservative patients. In the Teernstra trial 25% of the 16 surgical patients and 22% of the 9 conservative patients had a favourable outcome. Thus overall 42% of surgical patients and 27% of conservative patients had a favourable outcome. Figures 2a and 2b show meta-analyses of lobar haematomas from these trials excluding and including the STICH study data.

Therefore the few published randomised controlled trial data that did exist concerning lobar haematomas supported the hypothesis that this subgroup might benefit from early surgery.

An unfortunate outcome of STICH had been that many people misinterpreted the results to argue that there was no need to operate on patients with ICH at all. However neurosurgeons know that early removal of an intracranial haemorrhage is highly effective postoperatively and in the context of trauma (extradural haematoma [20], and acute subdural haematoma [21]). It seems unlikely that surgery would be of benefit in one scenario and not in the other. To leave patients with lesions that should be removed (an unfortunate misinterpretation of STICH) would condemn such patients to non-operative treatment perhaps for evermore. Since STICH was not powered sufficiently to answer the question about this subgroup alone there was an urgent need to undertake STICH II.

### STICH II will establish whether surgery is of benefit to a sub group

The trial aims to establish whether a policy of earlier surgical evacuation of the haematoma in selected patients with spontaneous lobar ICH will improve outcome compared to a policy of initial conservative treatment. The trial will also help to better define the indications for early surgery.

This will overcome two of the criticisms of STICH (timing was too late and sometimes location was too deep). The subgroup identified in STICH is clinically sensible and the hypothesis identified for STICH II is in line with current neurosurgical opinion.

### Design

STICH II is an international multicentre randomised parallel group trial comparing early craniotomy to evacuate the haematoma with initial conservative treatment, following spontaneous superficial intracerebral haemorrhage affecting the lobar region only. Only patients for whom the treating neurosurgeon is in equipoise about the benefits of early craniotomy compared to initial conservative treatment are eligible for the trial. Outcome is measured at six months via a postal questionnaire including the Glasgow Outcome scale, Modified Rankin Scale, EuroQol and Barthel. Six hundred patients will be recruited to the trial.

### Centre eligibility

At least 100 centres from around the world are included (UK, USA, Australia, Armenia, Czech Republic, Egypt, Germany, Greece, Hungary, India, Italy, Latvia, Lithuania, Macedonia, Mexico, Nepal, Pakistan, Poland, Romania, Russia, Spain, and Turkey). Only centres that can demonstrate effective trial experience and previous adherence to trial guidelines with high follow-up rates are eligible to take part.

### Approval to start

MREC approval for the study was obtained from Scotland A Multicentre Research Ethics Committee. Appropriate local ethical approval is sought from each participating centre in the study with proof of the approval forwarded to the trial coordinating office before recruitment can be started. The trial is conducted according to local ethical and Research and Development procedures. An agreement is signed between the sponsor (Newcastle upon Tyne NHS Hospitals Foundation Trust), the holder of the study funding (Newcastle University) and the hospital centre prior to commencing the study at the centre.

### Inclusion Criteria

• Evidence of a spontaneous lobar ICH on CT scan (1 cm or less from the cortex surface of the brain).

• Patient within 48 hours of ictus.

• Best MOTOR score on the Glasgow Coma Scale (GCS) of 5 or 6 and best EYE score on the GCS of 2 or more.

• Volume of haematoma between 10 and 100 ml [Calculated using (a × b × c)/2 method].

### Exclusion Criteria

• Clear evidence that the haemorrhage is due to an aneurysm or angiographically proven arteriovenous malformation.

• Intraventricular haemorrhage of any sort.

• ICH secondary to tumour or trauma.

• Basal ganglia, thalamic, cerebellar or brainstem haemorrhage or extension of a lobar haemorrhage into any of these regions.

• Severe pre-existing physical or mental disability or severe co-morbidity which might interfere with assessment of outcome.

• If surgery cannot be performed within 12 hours.

• If the haematological effects of any previous anticoagulants are not completely reversed.

### Trial interventions

The trial intervention is early evacuation of the haematoma by the method preferred by the treating neurosurgeon, usually craniotomy, combined with appropriate best medical treatment versus best medical treatment, combined with delayed evacuation only if it becomes necessary later. In STICH 26% of patients crossed over from conservative treatment to surgery usually because of deterioration but information was scarce. This is a major problem with surgical trials and crossovers of this size are common [22]. In STICH II the aim is to have fewer crossovers and more detailed information about the reasons. Further information about the status (GCS and focal signs) of all patients through the first five days of their trial progress is collected in order to be able to monitor the change in status that leads to a change in equipoise for the treating neurosurgeon. All patients also have an additional CT scan at about five days (+/- 2 days) to assess changes in the haematoma size with and without surgery. This will enable the study to demonstrate the amount of clot removed by surgery.

### Allocation of patients and consent

All patients who are considered for STICH II must have a CT scan to confirm the diagnosis and the size and location of the haematoma. Any clotting or coagulation problems must be corrected.

Written witnessed informed consent of patients must be obtained prior to randomisation by trained neurosurgical staff. A member of neurosurgical staff must provide each patient and their relatives with a written information sheet about the study and allow as much time as possible to discuss the options. If the patient is unable to give consent themselves due to the nature of the haemorrhage, a personal representative must be approached to give assent on behalf of the patient. The personal representative is the person with the closest personal relationship with the patient who is themselves capable and willing to assent on behalf of the patient. If the patient is unable to consent and the closest relative is not available the patient cannot be included in the study. In Scotland, if proxy consent is necessary this is obtained from the welfare guardian or, if there is none, from the nearest relative.

One copy of the signed consent/assent form is given to the patient, one is filed in the patient notes and one is filed with the trial documentation.

This study does not permit assent from a professional representative or randomisation without prior consent/assent.

### Randomisation

It is not possible to blind either patients or treating surgeons to when the patient has had surgery or whether they have had surgery. To minimise possible sources of bias randomisation is undertaken centrally by an independent organisation (Centre for Healthcare Randomised Trials, Aberdeen). The allocation is stratified by country group with a minimisation procedure dependent on prognostic criteria and with a random element.

Randomisation must take place within 48 hours of ictus. Randomising clinicians complete a one-page randomisation form before contacting the central 24-hour randomisation service by telephone or web. The randomisation form records demographic and clot characteristics and status at randomisation. This information is required in order to randomise the patient.

During the randomisation process the neurosurgeon is informed of the treatment group the patient is allocated to plus the patient identifier number for the trial. The neurosurgeon records this information on the randomisation form and then faxes the form to the STICH Coordinating centre in Newcastle, UK.

Best medical treatment must begin as soon as possible and continue throughout follow-up, as required. If the patient is randomised to early surgery this should be undertaken within 12 hours of randomisation.

### Data Collection and Six Month Follow-up

The data manager at the STICH Coordinating centre in Newcastle, UK checks the information on the faxed randomisation forms against the information received from the randomisation service and enters the data into an anonymised password protected database. A list of patient names and study numbers is kept in a separate file to ensure patient confidentiality is maintained.

At two weeks after randomisation or at discharge or at death (whichever occurs first), the discharge/2 week form is completed by the responsible neurosurgeon or proxy. This form records:

• The event that triggers the form (i.e. death, transfer or discharge) and the patient's status at that time.

• Whether the patient has had surgery (and why if randomised to initial conservative treatment or why not if randomised to early surgery).

• The patient's GCS and localising features for the five days following randomisation.

• The occurrence of any adverse events following randomisation.

• Past medical history and status prior to the ictus.

• Glasgow Coma Score and Glasgow Outcome Scale at discharge from the neurosurgical unit or at two weeks (whichever is earlier).

These data are used by the Data Monitoring Committee to monitor progress of the trial.

The discharge/2 week form together with copies of the randomisation CT scan and the 5-day post randomisation CT scan are sent to the STICH office within two weeks. The preferred method of sending CT scans is in DICOM compatible format. DICOM images (on separate CDs for the two time points) are sent anonymised with patient identifier. The data manager enters the data into the anonymised password protected database. The CTs are analysed by trained readers blinded to treatment group and patient identity.

Postal follow-up occurs at six months. Structured postal questionnaires are used. They have been translated into the necessary languages. The patient's GP (in the UK) or consultant (outside the UK) is contacted at four months to confirm that the patient is alive, to confirm his/her place of residence and to request completion of the adverse events form. The six-month outcome questionnaire is mailed to the patient or carer at five months and followed with a reminder at six months if needed and telephone follow-up at seven months by "blinded" clerical or nursing staff, if necessary.

In countries where the postal system is problematic the patients are asked to attend a follow-up clinic where the questionnaires will be distributed and collected by an independent researcher. In countries where literacy or language/dialect is problematic an independent blinded interviewer administers the questionnaire. This same methodology was used successfully in STICH.

The aim is to achieve 100% follow-up and this can be achieved with the full cooperation of the centre investigators.

### Data storage

All paper copies of questionnaires are kept in locked filing cabinets in a locked office. All computerised data is password protected.

### Analysis

#### Outcome Measures

##### Primary

Unfavourable outcome will be death or severe disability which will be defined using a prognosis based 8 point Glasgow Outcome Scale/Modified Rankin Scale [17,19].

##### Secondary

Mortality, Rankin, EuroQol, Survival, living arrangements.

### Sample size

Subgroup analysis of the STICH trial demonstrated that for patients with only a lobar haematoma without an intraventricular extension 37% had a favourable outcome with initial conservative treatment and 49% had a favourable outcome with early surgery. With a 37% favourable outcome from conservative treatment a sample size of 566 would be required to show a 12% benefit from surgery (2p < 0.05) with 80% power. A sample size of 600 was chosen to allow for some loss to follow up and a small crossover rate.

### Blinding

The multidisciplinary team in the co-ordinating centre and the principal investigators are blinded to the results until after the data set is locked following receipt of the final outcome questionnaire. Only the data manager has access to unblinded data.

### Statistical analysis

Analysis will be on an "intention to treat" basis. The primary analysis will be a simple categorical frequency comparison using the chi-squared test for prognosis based favourable and unfavourable outcomes at six months [19,23]. Patients with a good prognosis will be categorised as having a favourable outcome if they achieve good recovery or moderate disability on the Glasgow Outcome scale. Patients with a poor prognosis will be categorised as having a favourable outcome if they achieve good recovery, moderate disability or upper severe disability on the extended Glasgow outcome scale. Logistic regression analysis will be undertaken to adjust for covariates. Secondary outcomes will also be analysed using the prognosis based method as specified in STICH [17].

Any subgroup analyses will be based on tests of interaction. The predefined subgroups include the following:

Age

Volume

Glasgow Coma Score

Time from ictus to randomisation

Severity of neurological deficit

Planned method of haematoma removal

### Data and Safety Monitoring

#### Roles and responsibilities of Data Monitoring Committee

The data monitoring committee considers data from interim analyses and reports to the Trial Steering Committee. Interim analyses are strictly confidential and the committee will only recommend stopping the trial early if one or other treatment shows an advantage at a very high significance level.

### Roles and Responsibilities

#### Roles and responsibilities of Principal Investigators and trial coordinating team

Professor A D Mendelow has overall responsibility for the trial.

Dr B A Gregson is responsible for the overall day-to-day conduct of the trial including availability of co-ordinating advice in Newcastle.

Professor G D Murray is responsible for overall statistical validity of the trial.

Mr P Mitchell is responsible for recruiting centres and for analysis and publication of results.

Dr A R Gholkar is responsible for the central reading of CT scans.

The data manager is responsible for maintaining computerised databases containing all data related to the trial, for the quality of computerised information, for conducting preliminary analyses and preparing reports for the Data Monitoring Committee, for providing information to the applicants and for preparing monthly newsletters.

The trial secretary is responsible for all trial correspondence in relation to the trial, for sending postal questionnaires and reminders, for the organisation of investigator meetings and travel for monitoring, maintaining telephone and fax communications, preparing quarterly newsletters and publications, and reimbursing centres.

#### Roles and responsibilities of National Investigators

In countries with multiple centres one centre investigator fulfils the role of National Investigator. National investigators are responsible for obtaining national ethical approval, for ensuring that documentation is translated from English as required, for identifying suitable centres within their country, for encouraging recruitment and acting as a liaison person between the STICH Coordinating centre team and the centre if required. Each National Neurosurgical Investigator should work with a nominated Physician Champion within their country in order to promote the trial to other colleagues.

#### Roles and responsibilities of Centre Investigators

Each centre agrees to follow the protocol. They provide and update the trial coordinating team with their full address and contact details as necessary. Within each centre there is at least one named collaborator who is responsible for the conduct of the trial in his/her centre and in particular for:

• local ethical applications

• disseminating information about the trial within the centre

• maintaining local trial documentation

• identifying suitable patients

• ensuring all case report forms are completed and returned to the STICH office in Newcastle expeditiously

• ensuring copies of CT scans are provided to STICH office in Newcastle expeditiously

• ensuring follow-up is obtained in the centre

• attending investigator meetings

• facilitating centre monitoring

• commenting on the final report

#### Roles and responsibilities of Trial Steering Committee

The Trial Steering Committee provides overall supervision of the trial on behalf of the MRC. It considers progress of the trial, adherence to the protocol, patient safety and consideration of new information. The trial is conducted according to the standards set out in the MRC Guidelines for Good Clinical Practice.

### Trial Organisation

#### STICH Co-ordinating Centre (for all information and queries)

STICH Office, Neurosurgical Trials Unit, Newcastle University, 3-4 Claremont Terrace, Newcastle upon Tyne, NE2 4AE

Website: http://research.ncl.ac.uk/stich/

Email: stich@ncl.ac.uk

Phone: +44 191 222 5761

Fax: +44 191 222 5762

### Randomisation Service

Aberdeen HSRU +44 (0) 1224 551 261

https://viis.abdn.ac.uk/HSRU/stich/

### Steering Committee

Professor P Sandercock (Independent Chairman)

Professor G Ford, (Independent Member)

Professor D Barer, (Host Institution Member)

Professor A Strong (Independent Member)

Professor AD Mendelow

Dr BA Gregson

Mr PM Mitchell

Dr AR Gholkar

Professor GD Murray

### Data Monitoring Committee

Professor D Hanley (Chairman)

Mr DT Hope

Dr A Skene

Miss Helen Fernandes

### Trial Management Team

Dr Barbara A Gregson (Trial Director)

Professor A David Mendelow (Chief Investigator)

Dr Elise Rowan (Data Manager 01/05/2008 -)

Dr Alina Andras (Data manager 01/08/2010 -)

Miss Gillian Kenyon (Trial Secretary)

### Sponsor

Newcastle upon Tyne Hospitals NHS Trust (Sponsor No: 3545).

### Funding Source

Medical Research Council (UK) now allocated to Efficacy and Mechanism Evaluation Programme of the National Institutes of Health Research (NIHR):

MRC Grant No: G0501444

EME Number: 09-800-18

This report is independent research funded by the MRC and managed by the NIHR on behalf of the MRC-NIHR partnership. The views expressed in this publication are those of the authors and not necessarily those of the MRC, NHS, NIHR or the Department of Health.

## List of Abbreviations

STICH: Surgical Trial in Intracerebral Haemorrhage; STICH II: Surgical Trial in Lobar Intracerebral Haemorrhage; ICH: Intracerebral Haemorrhage; CT: Computerised Tomography; IVH: Intraventricular Haemorrhage; MREC: Multicentre Research Ethics Committee (in the UK); NHS: National Health Service (in the UK); GCS: Glasgow Coma Scale; ml: Millilitres; cm: Centimetres; DICOM: Digital Imaging and Communications in Medicine; GP: General Practitioner; MRC Medical Research Council (UK); EME: Efficacy and Mechanism Evaluation Programme of the National Institutes of Health Research; NIHR: National Institutes of Health Research

## Competing interests

Professor A David Mendelow is a director of the Newcastle Neurosurgery Foundation Ltd and has received honoraria for attending advisory committee meetings for Codman, Stryker and Novo Nordisc.

## Authors' contributions

ADM, PMM and BAG are responsible for the conception of the study, protocol design and study coordination. GDM has provided statistical guidance throughout the study and assistance with initial study design. ARG coordinates and provides support with CT scan acquisition and analysis and has also been involved with study management. ENR has been involved with the development of the study. All authors have been involved in the drafting of the manuscript and have approved it prior to submission for publication.
